# Modelling the role of tourism in the spread of HIV: A case study from Malaysia

**DOI:** 10.1016/j.heliyon.2024.e35896

**Published:** 2024-08-08

**Authors:** Ofosuhene O. Apenteng, Philip Rasmussen, Beate Conrady

**Affiliations:** Department of Veterinary and Animal Sciences, University of Copenhagen, Denmark

**Keywords:** AIDS, Compartment model, HIV, Malaysia, Reproduction number, Tourism

## Abstract

This study aimed to assess the role of tourism in the spread of human immunodeficiency virus (HIV) using Malaysian epidemiological data on HIV and acquired immunodeficiency syndrome (AIDS) incidence from 1986 to 2004. A population-level mathematical model was developed with the following compartments: the population susceptible to HIV infection, the clinically confirmed HIV-positive population, the population diagnosed with AIDS, and the tourist population. Additionally, newborns infected with HIV were considered. Sensitivity analyses and variations in fixed parameter values were used to explore the effect of changes to various parameter values on HIV incidence in the model. It was determined that variations in the rate of HIV-positive inbound tourist entries and the rate of foreign tourist exits (i.e., the duration of time tourists spent in Malaysia) significantly impacted the predicted incidence of HIV and AIDS in Malaysia. The model's fit to observed HIV and AIDS incidence was evaluated, resulting in adjusted R^2^ values of 53.3% and 53.2% for HIV and AIDS, respectively. Furthermore, the reproduction number (R_0_) was also calculated to quantify the stability of the HIV endemicity in Malaysia. The findings suggest that a steady-state level of HIV in Malaysia is achievable based on the low value of $R_{0}$ = 0.0136, and the disease-free equilibrium was stable from the negative eigenvalues obtained, which is encouraging from a public health perspective. The Partial Rank Correlation Coefficient (PRCC) values between the proportion of newborns born HIV-positive, the rate of Malaysian tourist entries returning home after contracting HIV, and the rate of foreign tourist exits have a significant impact on the $R_{0}$. The methods provide a framework for epidemiological modelling of HIV spread through transient population groups. The model results suggest that the role of tourism should not be overlooked within the set of available measures to mitigate the spread of HIV.

## 1 Introduction

HIV is a dangerous infection that attacks the immune system [1,2]. The virus mostly targets immune system CD4^+^ T cells [3], which are central to achieving a regulated effective immune response to pathogens [3]. HIV infection begins with little to no symptoms, and in the early months, it is characterized by slight changes to the immune system, until seroconversion occurs to the point where HIV-specific antibodies can be detected [4]. While the outcome of infection varies widely across individuals, the progression of the disease is typically slow [5], often taking several years to move from the primary infection stage to the symptoms of advanced HIV disease, immunosuppression [4], and increased vulnerability to co-infections [6]. Sexual transmission is the main pathway for the spread of HIV [7], with unprotected anal or vaginal sex greatly increasing the risk of infection [[8], [9], [10]]. Breastfeeding and pregnancy can also pass the virus from mother to child [11], with contaminated organ donations, infusions with contaminated blood or blood products, and injections with contaminated needles being additional transmission pathways [[12], [13], [14], [15]]. HIV-positive people are also at risk for persistent infections from other sexually transmitted infections (STIs), such as gonorrhea, chlamydia, herpes, and syphilis, while STIs increase HIV transmission efficiency through both an increased likelihood of transmission and an increased susceptibility to HIV infection [16,17]. Modelling of co-infection between HIV and Hepatitis C virus (HCV) has been investigated to understand how fractional calculus can be used to model infectious diseases [18].

Globally, tourism is among the fastest-growing industries [19] and revenue from this sector is an essential contributor to many economies, including Malaysia [20]. The Malaysian government began to take the expansion of the tourism industry seriously in the 1980s, with the establishment of The Ministry of Culture, Arts and Tourism in 1987, which later evolved into the Ministry of Tourism [21]. The role of tourism in Malaysia's economic well-being was further emphasized with the adoption of the nation's Third Industrial Master Plan (IMP3), which identified tourism services as a non-governmental sub-sector to be targeted for more significant development and promotion [21,22]. Tourism revenue increased from 4% of Gross domestic product (GDP) in the early 1990s to 16% in 2014. The contribution of tourism to the Malaysian economy has steadily increased, with almost 15% of the nation's total employment attributable to tourism-related activities [21,23]. The country saw more than a 140% increase in tourist arrivals during the same period. Since the year 2000, Malaysia's global tourism ranking has improved, rising from 17th place in 2000 to 10th place in 2012 [21,24]. In addition, over 27 million foreign tourists visited Malaysia in 2016, bringing in USD23 billion from tourism-related activities [23]. However, during the COVID period, tourist entries to Malaysia dropped to 4.3 million in 2020 in response to globally imposed restrictions on international travel [25], and the country's earnings from foreign tourism fell to USD3.0 billion that year [26]. However, in 2022, when travel restrictions eased, tourist entries rebounded to over 10 million [27].

Despite its critical economic role and associated benefits, it is vital to consider the potential for infectious disease transmission between tourists and the populations in their destination countries [28]. Several studies have suggested that touristic regions are associated with increased HIV risk among destination populations. For example, Ketshabile et al. (2007) investigated the relationship between tourism and HIV and the final stage of HIV, which is acquired immunodeficiency syndrome (AIDS), in southern Africa [29], and the role of tour guides/operators has been investigated [30]. Padilla et al. (2010) argued that the direction of future studies will be influenced by the change in the environmental perspective on sexual health in Caribbean tourist destinations and the incidence of HIV/AIDS cases [31]. By conducting a qualitative study of policymakers in the Dominican Republic, Guilamo-Ramos et al. (2013) explored the policy environment of prevention efforts regarding tourism-related HIV in the Caribbean [32]. A study by De Matos et al. (2013) suggested that, in Central Brazil, most sexually transmitted diseases have increased due to the sex trade through tourist-aimed prostitution [33]. In Malaysia, up to 70,000 new STIs are reported annually [34,35], and therefore, the sexual behavior of tourists can play a role in the transmission and spread of STIs and HIV, especially when unprotected sexual activity takes place [36,37]. A better understanding of the role of tourists within the transmission dynamics of HIV, particularly in a nation like Malaysia where tourism is a focal point of economic strategy, would benefit both human health and the economy.

It is known that mathematical models can help public health authorities plan for disease preparedness and mitigation measures [[38], [39], [40], [41]]. However, few mathematical models focus on the impact of tourism on HIV transmission. Some studies have investigated the role of tourists in spreading the disease [42,43], while others have used simulation models to determine the effectiveness of different approaches to controlling HIV transmission caused by tourism [[43], [44], [45]]. However, existing mathematical simulations have some limitations. They do not consider that children born to infected parents may be carriers of the disease, the birth rate within the susceptible population is independent of vertical transmission, and the study periods are not long enough. Therefore, this study aims to investigate the role of tourism in the spread of HIV with conderation for these omissions.

## 2 Materials and methods

### 2.1 Data

The epidemiological data from Malaysia between 1986 and 2004 was used to develop and validate a compartmental epidemiological model [46] (section 2.2). The data included the incidence of clinically confirmed HIV cases, the incidence of AIDS cases, and the number of tourist entries into Malaysia. The model was developed based on the reported data that three individuals acquired HIV in 1986, which made up the original HIV cases compartment I(0). Additionally, one person developed AIDS due to their HIV infection, making up the AIDS compartment A(0). The duration of data was between 1986 and 2004.The susceptible compartment S(0) had 16,329,396 members, which was the entire domestic population of Malaysia. The HIV compartment I(0) had three cases, and the AIDS compartment A(0) had only one case. In 1986, there were 3,200,000 tourist entries into Malaysia [47].

### 2.2 Description of the model

**Fig. 1:**
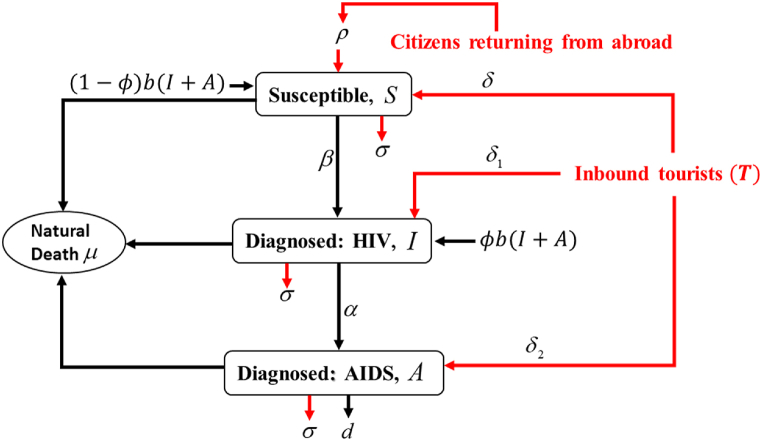
Schematic model for assessing tourism impact on HIV/AIDS incidence. S = population susceptible to HIV infection;I = population with clinically-confirmed HIV; A = population diagnosed with AIDS; ϕ = the proportion newborns born HIV-positive; b = the natural birthrate; (1−ϕ)b(I+A) = the fraction of newborns susceptible to HIV infection; β = the rate of susceptibility to HIV infection; μ = the natural death rate; d = AIDS-related death rate; α = the rate of population transfer from HIV-positive to AIDS-positive; δ = the rate of inbound tourist entries with susceptibility to HIV infection; $\delta_{1}$ = the rate of HIV-positive inbound tourist entries; $\delta_{2}$ = the rate of AIDS-positive inbound tourist entries; σ = the rate of foreign tourist exits; ρ = the rate of Malaysians returning home after contracting HIV abroad with unconfirmed infection status.

The study used a compartment model based on the SIR (susceptible, infectious, recovered/removed) framework (Fig. 1), commonly used in epidemiological modelling studies. The proposed HIV spread model in this study consists of four components: the susceptible population *S(t)*, the clinically confirmed HIV-positive population I(t), and the population diagnosed with AIDS A(t). Assuming that both I(t) and A(t) were sexually active, newborns who were HIV-positive joined the HIV compartment at a rate of b. The proportion of newborns born HIV-positive is represented by ϕ [48], while *b* is the natural birth rate. Hence, *(*1−ϕ)b(I+A*)* denotes the proportion of newborns susceptible to HIV infection. The rate of susceptibility to HIV transmission through sexual contact is denoted by β. It is assumed that the natural death rate μ remains constant across all compartments. The parameter *d* represents the rate of AIDS-related death, while the parameter α represents the rate of transfer from the HIV-positive compartment to the AIDS-positive compartment.

In a study by Apenteng (2017) [43], it was observed that tourists entered three different compartments at different rates. The compartments were susceptible, HIV-positive, and AIDS-positive. The rates of entry were defined as δ*,* $\delta_{1}$*,* and $\delta_{2}$, respectively, where δ is the rate of susceptibility of tourists, $\delta_{1}$ is the rate of HIV of the tourists, and finally*,* and $\delta_{2}$ is the rate of the AIDS of tourist. Similarly, tourists exited each compartment at a rate of *σ*, representing the rate of foreign tourist exits. The study also considered the proportion of Malaysian tourists who traveled abroad and contracted HIV before returning home, denoted as ρ. To estimate the population in each compartment, differential equation (1) through (3) below were used to calculate the rates of change.

(1) $$\frac{dS}{dt}=\delta T+\rho S+\left(1-\phi \right)b\left(I+A\right)-\beta \frac{\left(I+A\right)}{N}S-\left(\sigma +\mu \right)S$$

(2) $$\frac{dI}{dt}=\delta_{1}T+\phi b\left(I+A\right)+\beta \frac{\left(I+A\right)}{N}S-\left(\alpha +\sigma +\mu \right)I$$

(3) $$\frac{dA}{dt}=\delta_{2}T+\alpha I-\left(\sigma +d+\mu \right)A$$

### 2.3 Analysis of the model

In this section, we analyze the model and the equilibrium points with regard to the stability, instability of the model and the basic reproduction number.

#### 2.3.1 Equilibrium and stability analysis

We analyze the equilibrium points to find out whether there are changes or constants. To determine this disases free equilibrium, (1), (2), (3) becomes $\frac{dS}{dt}=\frac{dI}{dt}=\frac{dA}{dt}=0$*.* For information, see [[49], [50], [51]]

(4) $$\delta T+\rho S^{*}+\left(1-\phi \right)b\left(I^{*}+A^{*}\right)-\beta \frac{\left(I^{*}+A^{*}\right)}{N}S^{*}-\left(\sigma +\mu \right)S^{*}=0$$

(5) $$\delta_{1}T+\phi b\left(I^{*}+A^{*}\right)+\beta \frac{\left(I^{*}+A^{*}\right)}{N}S-\left(\alpha +\sigma +\mu \right)I^{*}=0$$

(6) $$\delta_{2}T+\alpha I^{*}-\left(\sigma +d+\mu \right)A^{*}=0$$

From (6), we obtain,

(7) $$A^{*}=\frac{\delta_{2}T+\alpha I^{*}}{\left(\sigma +d+\mu \right)}$$

by substituting (7) into (4) and (5), respectively, and by making solving for $S^{*}$ and $I^{*}$ we obtain

$$S^{*}=\frac{\left(\sigma +d+\mu \right)\left(\alpha +\sigma +\mu \right)N-\left(\sigma +d+\mu \right)\delta_{1}TN-\phi b\delta_{2}TN-\left(\sigma +d+\mu \right)\phi bTN-\phi b\alpha TN}{\left(\sigma +d+\mu \right)\beta +\beta \alpha N+\beta \delta_{2}TN}$$

$$I^{*}=\frac{\left(\sigma +d+\mu \right)\delta TN+\rho S^{*}N\left(\sigma +d+\mu \right)-\left(1-\phi \right)b\left(\sigma +d+\mu \right)\delta_{1}TN-\beta S\delta_{2}T-\left(\sigma +d+\mu \right)\left(\sigma +\mu \right)N}{\left(\sigma +d+\mu \right)\beta +\beta \alpha N+\beta \delta_{2}TN}$$

The inflow of tourists suggested by (1), (2), (3) does not show a disease-free equilibrium. The endemic equilibrium $E^{*}=\left(S^{*},I^{*},A^{*}\right)$ occurs whenever there is HIV and AIDS found in the population. Now, from (2), the presence of $E^{*}$ is shown below

(8) $${F\left(S\right)=\delta }_{1}T+\phi b\left(I+A\right)+\beta \frac{\left(I+A\right)}{N}S-\left(\alpha +\sigma +\mu \right)I$$

Now from (8), $S\leq \frac{\delta T}{\mu +\sigma -\rho }$ has a positive root range between 0 and $\frac{\delta T}{\mu +\sigma -\rho }$. By substituting when S=0, (5) becomes:

(9) $${F\left(S\right)=\delta }_{1}T+\phi b\left(I+A\right)‐\left(\alpha +\sigma +\mu \right)I$$

and when $S=\frac{\delta T}{\mu +\sigma -\rho }$, (8) gives:

(10) $$F\left(\frac{\delta T}{\mu +\sigma -\rho }\right)=\frac{dI}{dt}=\delta_{1}T+\phi b\left(I+A\right)+\beta \frac{\left(I+A\right)}{N}\left(\frac{\delta T}{\mu +\sigma -\rho }\right)\;-\left(\alpha +\sigma +\mu \right)I$$

Now, when we differentiate (8) with respect to *S*, the final solution becomes:

(11) $$\frac{dF\left(S\right)}{dt}=\beta \frac{\left(I+A\right)}{N}$$

#### 2.3.2 Local stability of the equilibrium points

To determine the local stability of the endemicity of the disease, we use the eigenvalues of the characteristic equation of the Jacobian matrix for (1), (2), (3), $J\left(S^{*},I^{*},A^{*}\right)=J\left(E^{*}\right)$ This is given by:

(12) $$J\left(E^{*}\right)=\left[\begin{array}{ccc} \rho -\beta \frac{\left(I+A\right)}{N}-\left(\sigma +\mu \right) & \left(1-\phi \right)b-\beta \frac{S}{N} & \left(1-\phi \right)b-\beta \frac{S}{N}\\ \beta \frac{\left(I+A\right)}{N} & \phi b+\beta \frac{S}{N}-\left(\alpha +\sigma +\mu \right) & \phi b+\beta \frac{S}{N}\\ 0 & \alpha & -\left(\alpha +d+\mu \right) \end{array}\right]$$

The $J\left(E^{*}\right)$ corresponding equation of the characteristic polynomial equation is given by

(13) $$f\left(\lambda \right)=\left(\lambda_{1}-\rho +\beta \frac{\left(I+A\right)}{N}+\left(\sigma +\mu \right)\right)\left(\lambda_{2}-\phi b-\beta \frac{S}{N}+\left(\alpha +\sigma +\mu \right)\right)\left(\lambda_{3}+\left(\alpha +d+\mu \right)\right)=0$$

where

$$\lambda_{1}=\rho -\beta \frac{\left(I+A\right)}{N}-\left(\sigma +\mu \right)\text{,}$$

$$\lambda_{2}=\phi b+\beta \frac{S}{N}-\left(\alpha +\sigma +\mu \right)\text{,}$$

$$\lambda_{3}=-\left(\alpha +d+\mu \right)\text{.}$$

### 2.4 Calculation of the reproduction number

The basic reproduction number $R_{0}$ (i.e., epidemiologic metric used to describe the contagiousness or transmissibility of infectious agents), is the anticipated number of new HIV-positive cases that a typical infected person introduces into a group with a specific percentage of protection [52,53]. The model's parameters and variables were assumed to be non-negative. From equation (1) through (3), when the disease is absent at equilibrium equation (1) becomes $S\left(t\right)=\frac{\delta T}{\mu +\sigma -\rho }$. However, under the dynamic restrictions of (1) through (3), the region $\Sigma =\left\{\left(x=S,I,A\right)\in R_{+}^{3}:S\leq \frac{\delta T}{\mu +\sigma -\rho }\;\right\}$ is positively invariant. So, for the initial starting point $x\in R_{+}^{3}$, the trajectory lies in Σ. The analysis in the current study is limited to this region Σ. The existence, uniqueness, and continuation results for equation (1) through (3) in this region are described in other studies [[54], [55], [56], [57], [58]]. From this, we now write the following two matrices:

(14) $$F=\left(\begin{array}{cc} \beta \frac{S}{N} & \beta \frac{S}{N}\\ 0 & 0 \end{array}\right)$$

(15) $$V=\left(\begin{array}{cc} -\phi b+\left(\alpha +\sigma +\mu \right) & -\phi b\\ -\alpha & \left(\alpha +d+\mu \right) \end{array}\right)$$

The inverse multiplication of (14) and (15) will give the spectral radius of the matrix of the basic reproduction number as $\frac{\beta \frac{S}{N}\left(\alpha +d+\mu \right)}{\left(\alpha +d+\mu \right)\left(\alpha +\sigma +\mu -\phi b\right)-\phi \alpha b}$ at equilibrium $S\left(t\right)=\frac{\delta T}{\mu +\sigma -\rho }$.

Therefore, the final basic reproduction number is

$$R_{0}=\frac{\beta \frac{\delta T\left(\alpha +d+\mu \right)}{N\left(\mu +\sigma -\rho \right)}}{\left(\alpha +d+\mu \right)\left(\alpha +\sigma +\mu -\phi b\right)-\phi \alpha b}$$

### 2.5 Model calibration

The model's parameters were calibrated to reflect Malaysian epidemiological data on HIV and AIDS incidence. Assume that θ represents the epidemiological parameters estimated and that θ is the independent and additive Gaussian prior. Let ϕ be the error, such that $\phi ∼N\left(0,\sigma^{2}\right)$. The Delayed-Rejection Adaptive Metropolis algorithm [59] was used to solve for θ, the posterior distribution of the defined model. The standard errors of the fitted parameters were approximated by taking the square root of the diagonal of the generalized inverse of the Hessian matrix of the fitted parameters [59]. Lastly, the model accuracy was evaluated by calculating the adjusted R^2^ [60,61] using the following equations:

(16) $$R^{2}=1-\frac{\sum_{i=1}^{n}{\left(y_{i}-{\hat{y}}_{i}\right)}^{2}}{\sum_{i=1}^{n}{\left(y_{i}-{\bar{y}}_{i}\right)}^{2}}$$

and

(17) $$R_{adj}^{2}=1-\left(1-R^{2}\right)\left(\frac{n-1}{n-p}\right)$$

where, ${\hat{y}}_{i}$, ${\hat{y}}_{i}$, and ${\bar{y}}_{i}$ represent the observed data values, the predicted data values, and the mean of the observed data values, respectively, and n and p represent the number of data points and the number of independent variables.

### 2.6 Sensitivity analysis

Using the package 'Flexible Modelling Environment' (FME) [59] in R programming language [62] within RStudio, sensitivity analyses were conducted to identify the parameters whose values significantly impacted the model's predictions. Specifically, the *sensFun* function within the 'FME' package estimates the sensitivity of the model's outputs to parameter values within a set of sensitivity functions [59]. These functions estimate the instantaneous rate of change of the model's output relative changes to the value of its parameters. As described in another study [59], the function generated a matrix of normalized, dimensionless sensitivities whose ${\left(i,j\right)}^{th}$ element $S_{ij}$ contains the following:

(18) $$\frac{\partial_{y_{i}}}{\partial \Theta_{j}}*\frac{w_{\Theta_{j}}}{w_{y_{i}}}$$

where, $y_{i}$ is an output variable and $\Theta_{j}$ is a parameter, and $w_{y_{i}}$ and $w_{\Theta_{j}}$ are the scaling weights of $y_{i}$ and $\Theta_{j}$, respectively [58]. The *L1* and *L2* norms, where the *L1* norm is defined as the sum of the absolute values of the vector (i.e., $L1=\sum \left|S_{ij}\right|/n$), and the *L2* norm is defined as the square root of the sum of the squared vector values (i.e., $L2=\sqrt{{(S}_{ij}^{2})/n}$), can then be compared across parameters to determine the relative sensitivity of the model's outputs to variations in the parameter values. Specifically, the larger the magnitude of these norms, the more significant the impact of variations in the parameter's value on the model's output. Additionally, the effects of changes in β and $\delta_{1}$ on the spread of HIV were explored by comparing the number of diagnosed cases of HIV from the period 1986 to 2004 across repeated simulations using a range of values for these parameters.

To determine which of these parameters affects the $R_{0}$, we subsequently conducted a sensitive analysis based on the $R_{0}$ for each estimated parameter. This was done by performing the Partial Rank Correlation Coefficient (PRCC) by calculating the PRCC values for each estimated parameter based on the $R_{0}$ [63,64].

## 3 Results

**Fig. 2:**
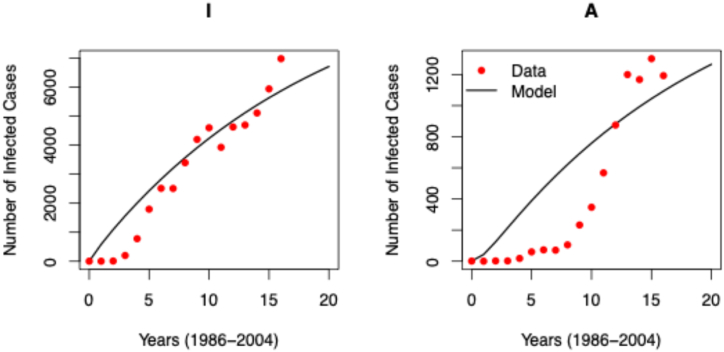
Reported cases of human immunodeficiency virus (HIV), corresponding to the Infected compartment (left), and acquired immunodeficiency syndrome (AIDS), corresponding to the AIDS compartment (right) in Malaysia (1986–2004) compared to the results of the fitted compartment model. Observations are shown in red while fitted model is shown in a solid black line.

The model was fitted to the Malaysian data, resulting in, among other estimates, an estimated HIV transmission rate of 4.767x10^−1^ per susceptible individual, a rate of compartmental transfer from HIV-positive to AIDS-positive of 1.885x10^−1^ per HIV-positive individual, and an exit rate of foreign tourists of 5.208x10^−1^ (Table 1). The epidemiological data indicates that HIV incidence in Malaysia peaked around 2001, with a subsequent decline, followed by a peak in AIDS incidence in 2004. The model's fitted parameters reflect these epidemiological data (Fig. 2) with adjusted R^2^ values of 53.3% and 53.2% for HIV and AIDS, respectively.

**Table 1:** Estimated parameters for the compartmental model based on Malaysian human immunodeficiency virus (HIV), acquired immunodeficiency syndrome (AIDS), and tourism data from 1986 to 2004.

Parameters	Definition	Estimate (95%CI)
β	Transmission rate	4.767x10^−1^ (4.735x10^−1^ - 4.798x10^−1^)
α	Rate of population transfer from HIV-positive to AIDS-positive	1.086x10^−1^ (1.081x10^−1^ - 1.092x10^−1^)
μ	Natural death rate	4.56x10^−3^ (Source: [65])
σ	Rate of foreign tourist exits	5.208x10^−1^ (5.176x10^−1^ - 5.239x10^−1^)
δ	Rate of inbound tourist entries with susceptibility to HIV infection	9.964x10^−1^ (Source: [46])
$\delta_{1}$	Rate of HIV-positive inbound tourist entries	1.992x10^−3^ (Source: [46])
$\delta_{2}$	Rate of AIDS-positive inbound tourist entries	1.992x10^−8^ (Source: [46])
ρ	Rate of Malaysian tourist entries returning home after contracting HIV abroad	9.837x10^−9^ (9.425x10^−9^ - 9.946x10^−9^)
d	AIDS-related death rate	1.23x10^−4^ (Source: [66])
b	Natural birth rate	2.91x10^−3^ (Source: [65])
ϕ	The proportion of newborns born HIV-positive	4.429x10^−6^(3.257x10^−5^ - 4.143x10^−5^)

Table 2 presents the results of sensitivity analyses using L1 and L2 norms. Based on both norms, the predicted number of HIV cases was found to be most sensitive to changes in α, the rate of population transfer from HIV-positive to AIDS-positive, and σ, the rate of foreign tourist exits (i.e., the duration of time foreign tourists spends in Malaysia). The parameter with the next highest impact was β, followed by ρ, the rate of Malaysian tourist entries returning home after contracting HIV abroad, which had the least impact.

**Table 2:** *L1* and *L2* norms were calculated to determine the sensitivity of the compartmental model's predicted number of human immunodeficiency virus (HIV) cases to variations in parameter values.

Parameter	*L1* norm	*L2* norm
β	1.00x10^−3^	1.50x10^−3^
α	3.30x10^−1^	5.10x10^−1^
σ	2.20x10^−1^	2.60x10^−1^
ρ	5.10x10^−9^	1.00x10^−8^
ϕ	7.10x10^−3^	1.00x10^−2^

According to Fig. 4 and Table 3, if the baseline foreign tourist exit rate (σ) is increased by 50%, there will be an estimated 218,377 cumulative HIV cases in the Malaysian population. This is equivalent to a ten-fold increase from the current estimated number of HIV cases, which is 82,255.

**Table 3:** The effect of variations in $\delta_{1}$, the rate at which HIV-positive tourists entered the Malaysian population, and σ, the rate of foreign tourists exits, on the cumulative predicted number of HIV cases in Malaysia between 1986 and 2004 based on the compartmental model.

*Changes in*$\delta_{1}$	HIV cases
No change (baseline)	82,255
50% increase	122,234
40% decrease	66,059
60% decrease	49,743
80% decrease	33,303
*Changes in*σ	*HIV cases*
No change (baseline)	82,254
50% increase	218,377
40% decrease	273,012
60% decrease	123,452
80% decrease	48,475

Conversely, a reduction in σ translates to a significant decrease in the predicted number of HIV cases, with, for example, an 80% decrease from the baseline σ resulting in 48,475 predicted cases of HIV, a decrease of over 50% in the number of HIV cases. Similarly, the effects of $\delta_{1}$ on the number of predicted HIV cases is also shown in Fig. 4. A 50% increase in $\delta_{1}$ will result in 122,234 new cases of HIV in the Malaysian population compared to the baseline prediction.

Table 4 and Fig. 5 can be utilized to determine the most significant estimated parameters for the basic reproduction number output, considering the PRCC values. The bigger the absolute PRCC value, the greater the impact of the parameter on $R_{0}$.

**Table 4:** Partial Rank Correlation Coefficients (PRCC) of the estimated parameters with $R_{0}$.

Parameters	Definition	PRCC values
β	Transmission rate	0.304
α	Rate of population transfer from HIV-positive to AIDS-positive	0.023
σ	Rate of foreign tourist exits	−0.355
ρ	Rate of Malaysian tourist entries returning home after contracting HIV abroad	−0.445
ϕ	The proportion of newborns born HIV-positive	0.391

## 4 Discussion and conclusions

**Fig. 3:**
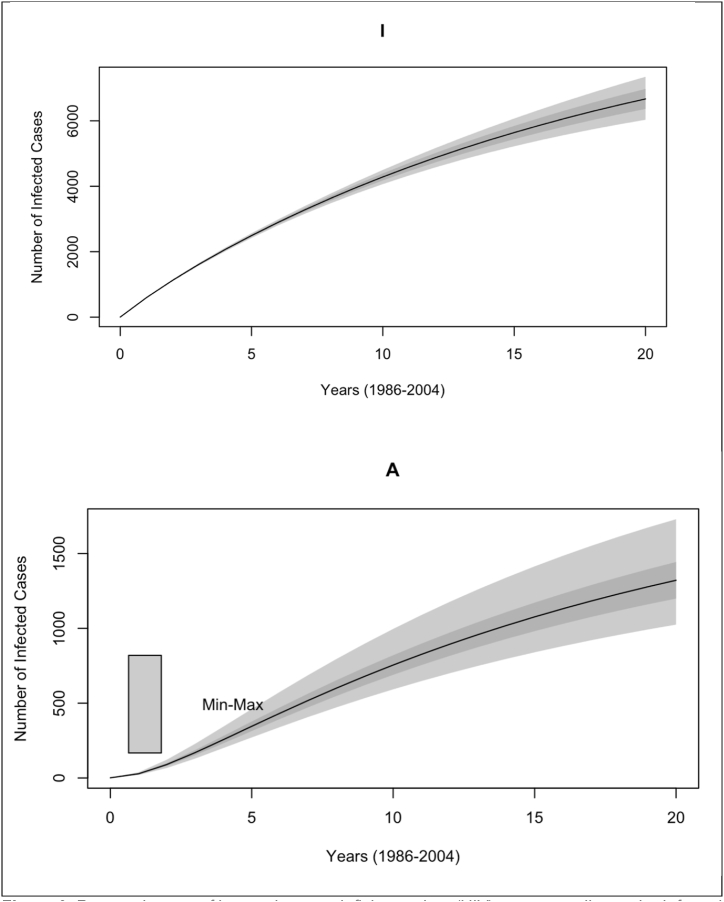
Reported cases of human immunodeficiency virus (HIV), corresponding to the Infected compartment (top) and acquired immunodeficiency syndrome (AIDS), corresponding to the AIDS compartment (bottom) in Malaysia (1986–2004), where the light gray shade represents the upper confidence interval and dark gray shade represents the lower confidence interval (Min-Max).

A population-level mathematical model was developed to investigate the role of tourism in the spread of HIV in Malaysia and fitted to epidemiological data on Malaysian HIV and AIDS with explicit consideration for newborns born with HIV. It was determined that variations in the model's tourism-related parameters, such as the rate of foreign tourist exits, which reflects the duration of touristic visits, and the rate of HIV-positive inbound tourist entries, which reflects the overall rate of touristic entries, significantly impacted the predicted cumulative incidence of HIV in Malaysia (Table 2, Table 3, and Fig. 3). Understanding the role of tourism in the spread of infectious diseases is crucial for national efforts to control or prevent the spread of HIV, particularly for countries that rely heavily on tourism, such as Malaysia. Additionally, this research suggests that a steady-state level of HIV in Malaysia is achievable, from a disease transmission perspective, with an estimated basic reproduction number $R_{0}<1$.

**Fig. 4:**
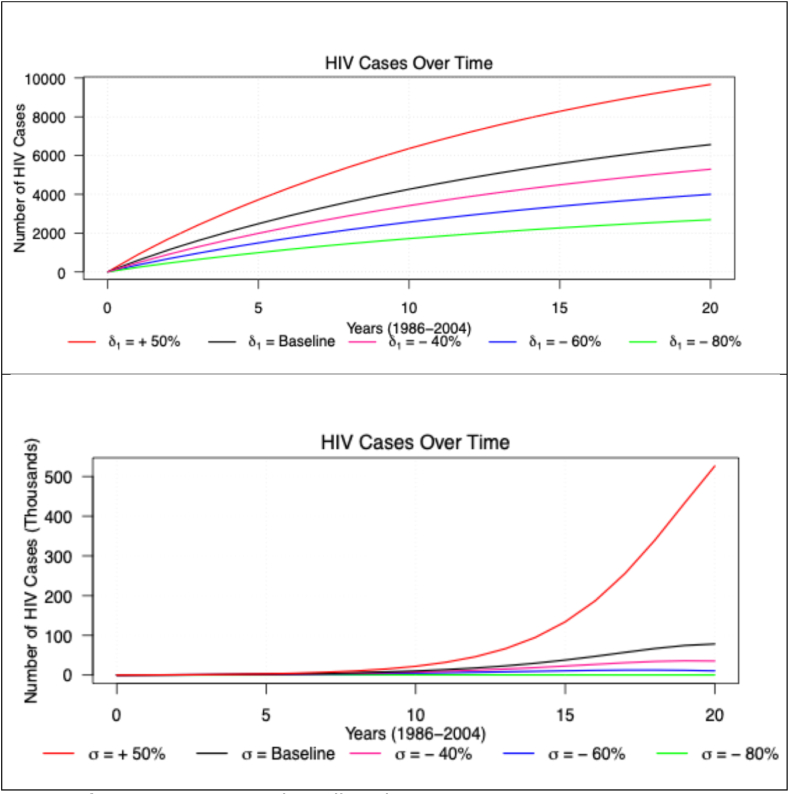
Graphical comparison of the effect of variations in $\delta_{1}$, the rate at which tourists moved to the HIV-positive compartment (top) and σ, the rate of foreign tourist exits (bottom) on the predicted number of HIV cases in Malaysia between 1986 and 2004 based on the compartmental model.

**Fig. 5:**
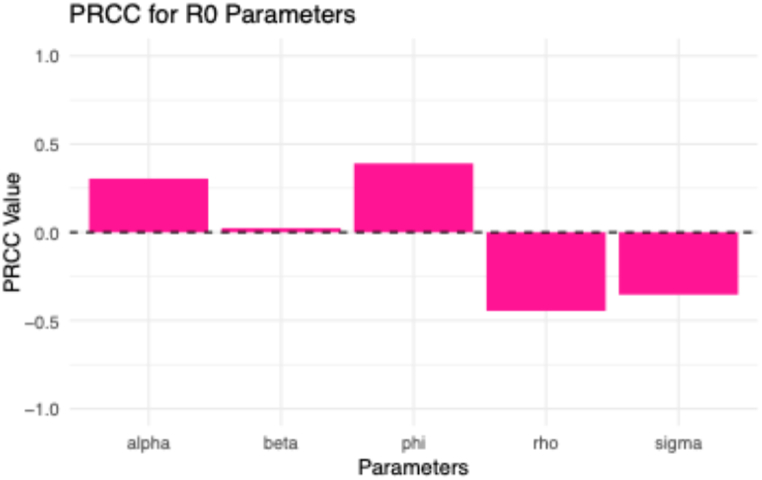
PRCC for the basic reproduction number with each of the estimated parameters.

Mathematical modelling is a valuable tool in researching different aspects of HIV [[67], [68], [69]], such as transmission between sexual partners [[70], [71], [72]] and intravenous drug users [73], as well as cell-to-cell spread [74]. It can also help prevent drug-resistant HIV and aid in the study the infection's pathogenesis and treatment [75]. Compartmental models are particularly relevant to this study's results, as they extend the work of Apenteng and Ismail (2014) and Apenteng (2017) by incorporating the proportion of newborns infected with HIV (as shown in Table 1) [42,43].

Specifically, by expanding on the Malaysian compartmental HIV model [42,43] to include the proportion of newborns ϕb(I+A) and, thus, the proportion of newborns born with HIV (1-ϕ)b(I+A), the current model more accurately reflects HIV transmission dynamics and yields different parameter estimations. For example, in the study by Apenteng (2017) [43], the estimated parameters for the rate of inbound tourists entering the susceptible, HIV, and AIDS compartments were 9.98x10^−2^, 3.19x10^−5^, and 5.86x10^−2^, respectively (Table 1).

This study proposes a new method to evaluate the impact of tourism on HIV transmission. The framework used in this method also estimates epidemiological parameters that can help develop surveillance and control efforts by identifying the factors that strongly influence HIV transmission. Based on the parameter vector's L1 and L2 norms, the sensitivity analyses suggested that the rate of foreign tourist exits (σ) was the most sensitive factor, meaning that the duration of time that foreign tourists spend in Malaysia has a significant impact on the predicted number of HIV cases. Despite identifying the potential role of touristic flows in HIV transmission, the formulation of an appropriate policy response is complex and may remain difficult to achieve. This complexity is apparent in compartmental models of HIV transmission in other regions and countries.

Positive PRCC values signify the positive correlation between an estimated parameter and $R_{0}$, as seen with ∅,α, and β. In contrast, negative PRCC values signify the negative correlation between an estimated parameter and $R_{0}$ , as seen with ρ and σ (for more details, see Table 4 and Fig. 5). These PRCC values give information on potentially effective mitigation plans to prevent the spread of HIV and AIDS. To determine the eigenvalues, we substituted the values of the estimated parameters and the initial values of the compartments; we have $\lambda_{1}=-0.5253$, $\lambda_{2}=-0.2372$ , and $\lambda_{3}=-0.5254$ from (13). This shows that the disease-free equilibrium is stable since all the eigenvalues have negative parts or values [76].

As an example, Nyabadza and Mukandavire (2011) developed a compartmental model in 2011 to predict the trend of HIV transmission in South Africa. They used simulations to fit their model [38], similar to the approach described in this paper. Their findings suggested that an individual's knowledge about HIV/AIDS, which is typically obtained through treatment provided during HIV testing and screening, can help reduce the likelihood of transmission. However, based on a further exploration of the impact of screening and treatment on the dynamics of HIV transmission in South Africa, the same authors suggested that, despite 1.6 million people undergoing HIV screening annually, screening and surveillance efforts alone will not stop the spread of HIV. Further, Nyabadza and Mukandavire (2011) estimated that the HIV epidemic in South Africa had an $R_{0}$ value of less than one, which means that the disease could not spread rapidly. However, they also noted that HIV could continue to spread without bounds unless there is a change in sexual behaviour in the country. To prevent new HIV infections, the authors suggested that people should reduce the number of sexual partners, use condoms, educate individuals about HIV, and change their sexual conduct overall [46]. This current study suggests that a steady-state level of HIV in Malaysia is achievable, from a disease transmission perspective, with an estimated reproduction number $R_{0}$ of 0.0136 (Table 1). In epidemiological terms, it is expected that transmission will wane and eventually stop when $R_{0}\leq 1$ and conversely, disease transmission will persist or increase indefinitely when $R_{0}>1$ [54,77]. It has been observed that HIV and AIDS are prevalent diseases in Malaysia. However, taking preventive measures such as reducing the number of sexual partners and using condoms can help in phasing out the disease. Nevertheless, it is essential to consider tourism as an ongoing risk for introducing diseases, as demonstrated in this study.

Bacaër et al. (2006) created a model to study the HIV/AIDS epidemic among injectable drug users and sex workers in Kunming, Yunnan, the provincial capital of China [40]. They developed a compartmental model in which individuals went through three stages, namely susceptible S, HIV-positive-AIDS-negative I, and AIDS-positive A compartments. Interestingly, their estimated basic reproduction number was 32, which means that, despite their assumption that individuals in the AIDS compartment did not have contact with others, the public health system was far more unstable than the one modeled in the current study. Bozkurt and Peker (2014) proposed that educating individuals about HIV and the risks of engaging in unsafe sexual behavior or any other risky behavior that increases the risk of HIV transmission would have the most significant impact on lowering the infection rate and prevalence [68]. Their compartmental model suggested that immigration is positively related to the spread of the disease. At the same time, a combination of alerting individuals of their HIV-positive status through screening and contact tracing can result in increased abstinence and, therefore, a reduction in spread. However, if contact tracing is omitted, the spread will increase regardless of status notification through testing and screening.

Naresh et al. (2008) conducted a study to understand the impact of HIV-positive immigration on different population sizes. Their research primarily focused on how the disease spreads through sexual intercourse and assumed that all infected individuals eventually progress to the AIDS compartment. Their findings suggested that, by limiting the number of sexual partners, the transmission of the disease can be reduced over time. In other words, the study highlights the importance of taking preventive measures to control the spread of HIV, especially in communities with high-risk factors. Benotsch et al. (2006) studied visitors to a popular tourist destination in the US. They found that these travelers were at a higher risk of contracting HIV and could potentially spread the virus to their home populations [78]. Zöldi et al. (2017) performed a non-compartmental study from 1995 to 2015 in Finland and examined all cases of STIs related to foreign travel. The authors discovered that out of approximately 9,000 STI cases, 2,300 (26%) were linked to travel. The study estimated the total risks and analyzed trends across different geographical areas [79].

Tourism is an essential source of income for many countries around the world. Therefore, when developing public health policies to prevent the spread of diseases, it is crucial to take into account the role of tourism. Some countries, such as the United States, Canada, Australia, New Zealand, Spain, Croatia, Denmark, and others, require visitors to undergo a medical examination before entering their territory, the requirement being based on the length of stay. This policy is valid as this study suggests that the incidence of HIV and other transmissible diseases is related to the duration of stay (σ or the rate of foreign tourist exit). Recently, Selvanathan et al. (2021) found that tourism significantly contributed to the spread of COVID-19 within and across countries due to the movement of individuals [80]. The COVID-19 pandemic resulted in the widespread adoption of travel restrictions [81], reflecting the vital role of transient populations such as international travelers and tourists [82,83]. Travel restrictions may be effective in reducing the transmission of HIV, however, imposing travel restrictions based on an individual's HIV status may be unethical. Therefore, public health policy should focus on developing and promoting education initiatives aimed at reducing the transmission of HIV among at-risk populations. In addition, comprehensive surveillance and contact tracing capacities should be developed to control the transmission of HIV further.

Finally, it is important to recognize the limitations of this study. We had to estimate the rate of Malaysians returning home after contracting unconfirmed HIV abroad, as well as the rate of foreign tourist exits since the actual values of these parameters were not available in the literature or the epidemiological data used in this study. The findings of this study may be negatively impacted by the lack of comprehensive epidemiological data from the Malaysian HIV outbreak up to this point. This is because the estimations of the parameters were affected by the amount of noise in the data [84,85]. Furthermore, while the average number of days spent in Malaysia was incorporated into the proposed model as a fixed parameter, the actual period a tourist stays in Malaysia may vary significantly in real-world conditions. Nevertheless, the parameters δ, $\delta_{1}$, $\delta_{2}$, and σ indicate that tourism had a substantial impact on HIV transmission in Malaysia from 1986 to 2004 (Table 3), based on the available data, and it is possible that by collecting more observations, we can improve the model's accuracy.

The study highlights the need for the country to take significant steps in quantifying the trend and magnitude of tourism. Developing platforms that will enable public health officials to implement effective control strategies is also crucial. The study highlights the need for the country to take significant steps in quantifying the trend and magnitude of tourism. It is also crucial to develop platforms that will enable public health officials to implement effective control strategies. The findings of this study suggest that visitors to Malaysia have played a role in the spread of HIV and AIDS infection among the Malaysian population from 1986 to 2004. The findings suggest that a steady-state level of HIV in Malaysia is achievable based on the low value of $R_{0}$ = 0.0136, which is encouraging from a public health perspective. Moreover, the modelling framework can be used to explore the roles of other population groups and transmission parameters. For instance, future modelling studies should consider factors such as engaging in sexual activity with multiple partners and not protecting oneself against HIV.

## Data availability statement

Data supporting the results presented in this manuscript is available as stated above.

## CRediT authorship contribution statement

**Ofosuhene O. Apenteng:** Conceptualization, Data curation, Formal analysis, Methodology, Software, Supervision, Validation, Visualization, Writing – original draft, Writing – review & editing. **Philip Rasmussen:** Conceptualization, Validation, Visualization, Writing – review & editing. **Beate Conrady:** Validation, Visualization, Writing – review & editing.

## Declaration of competing interest

All authors declared that no competing interests exist.
